# Assessment of Smear-Positive Pulmonary Tuberculosis and Associated Factors among Patients Visiting Health Facilities of Gedeo Zone, Southern Ethiopia: A Cross-Sectional Study

**DOI:** 10.1155/2023/2502314

**Published:** 2023-03-31

**Authors:** Bahru Mantefardo, Gizaw Sisay, Ephrem Awlachew

**Affiliations:** ^1^School of Medicine, College of Medicine and Health Sciences, Dilla University, Ethiopia; ^2^School of Public Health, College of Medicine and Health Sciences, Dilla University, Ethiopia; ^3^Department of Medical Laboratory, College of Medicinal Health Science, Dilla University, Ethiopia

## Abstract

**Introduction:**

Tuberculosis (TB) was one of the top causes of ill health and the leading cause of deaths worldwide until the coronavirus (COVID-19) pandemic. Hence, this study is aimed at assessing the prevalence of sputum smear-positive TB and associated factors among TB-suspected patients attending in Gedeo Zone health facilities, Southern Ethiopia.

**Methods:**

A facility-based cross-sectional study was conducted among 220 TB-suspected patients in Gedeo Zone health facilities from July 01 to Sep 30, 2021. Patients were grouped as smear positive if one sputum out of two was positive or two sputum smears became positive. Various descriptive statistics were computed using the SPSS-25, and factors to smear positivity were identified by multivariable logistic regression analysis. Odds ratio at 95% CI and *p* values < 0.05 were considered as indicators of statistical association.

**Results:**

The overall prevalence of smear-positive TB in Gedeo Zone health facilities was 18.2%, which is significantly high, and the MTB detection rate of GeneXpert was 29.5%. Contact with a TB patient, cigarette smoking, and previously treatment for TB were factors significantly associated with smear-positive TB.

**Conclusion:**

The prevalence rate of smear-positive PTB in the study area was 18.2% and 29.5% by direct sputum AFB and sputum GeneXpert, respectively. As a result, we recommend intervention on the identified associated risk factors and further studies to ascertain risk factors and their magnitude at the community level.

## 1. Introduction

Tuberculosis (TB) is a communicable disease that is a major cause of ill health, one of the top 10 causes of death worldwide, and the leading cause of death from a single infectious agent (ranking above HIV/AIDS). TB is caused by the bacillus *Mycobacterium tuberculosis*, which spreads when people who are sick with TB expel bacteria into the air, for example, by coughing. About a quarter of the world's population is infected with *M. tuberculosis* [1].

Tuberculosis (TB) has remained a major global health problem. In 2021, it was one of the top causes of ill health and one of the leading causes of death worldwide. Until the coronavirus (COVID-19) pandemic, TB was the leading cause of death from a single infectious agent, ranking above HIV/AIDS [2].

Of the 4.8 million people diagnosed with pulmonary TB worldwide in 2020, 59% were bacteriologically confirmed. This was a slight increase from 57% (out of a total of 6.0 million) in 2019 [3].

WHO classifies Ethiopia as one of the high-burden countries for TB [3]. The national population-based TB prevalence survey conducted in 2010/2011 revealed that the prevalence of smear-positive TB among adults and all age groups was 108 and 63/10^5^ persons, respectively. The prevalence of bacteriologically confirmed TB was 156/10^5^ persons, and the prevalence for all forms of TB was 240/10^5^ persons in Ethiopia [4].

The recommended strategy to control TB in low-income countries, including Ethiopia where the majority of the TB cases occur, is to detect and promptly treat smear-positive cases [5]. It is known that delayed diagnosis results in more extensive disease and more complications and leads to higher mortality. It also leads to an increased period of infectivity in the community [6].

Southern Regional State is one of Ethiopia's high TB burden regions, with a case detection rate lower than the national average, 71% [7]. Various community-based and institutional-based research findings showed that case detection in Gamo Gofa was 19.4% [8]; in Shashogo woreda, Southern Ethiopia, it was 4.6% [9]; and in the Sidam region, it was 4.5% [10]. According to these investigations, the scale of the problem in Southern Ethiopia is high. TB prevalence status of both the public and private healthcare facilities of Gedeo Zone needs to be known to give appropriate and timely intervention. Therefore, the aim of this study was to assess the prevalence of smear-positive pulmonary TB and associated factors among patients visiting health facilities of Gedeo Zone, Southern Ethiopia.

## 2. Methods

### 2.1. Study Area and Period

The study was conducted at Gedeo Zone health facilities from July 30 to Sep 30, 2021. Gedeo Zone is located 369 km from Addis Ababa along the southern Addis Ababa-Moyale international road and 90 km from Hawassa (the capital city of the region) in South Nation Nationality and People Regional State (SNNPRS). The zone has a subhumid tropical climate. Gedeo Zone has eight districts. The zone has one referral hospital, three primary hospitals, 38 health centers, 146 health posts, 4 NGO clinics, and 17 reported private health facilities. Based on the Gedeo Zone health office report, the estimated total population of the zone in 2018 was 1,166,695. There are 38 health centers, three public hospitals, one private hospital, eight private higher clinics, and one referral hospital providing DOT services in the zone.

### 2.2. Study Design

A facility-based cross-sectional study was conducted to assess the prevalence of smear-positive pulmonary TB and associated factors among TB-suspected patients visiting health facilities (public and private) in Gedeo Zone, Southern Ethiopia. All patients who were suspected for TB at Gedeo Zone healthcare facilities were the source population, and all TB-suspected patients who visited the selected healthcare facilities of Gedeo Zone were the study population. Adults aged 18 years older and having a cough of two weeks or more were included, while severely diseased patients were excluded.

### 2.3. Sample Size Determination and Sampling Procedure

The sample size of this study was calculated using a single population proportion formula by considering the proportion of smear-positive TB [11], 95% confidence level, degree of precision of 4%, and a 10% nonresponse rate. The final sample size was 220. There were 59 total public and private health facilities offering TB diagnostic and treatment services in Gedeo Zone. Due to feasibility issues, from the total of 59 health facilities, ten were randomly selected through simple random selection technique. Thus, one public hospital, five health centers, one private hospital, and three private higher clinics were selected. The calculated sample size for each health facility was allocated proportionally based on previous TB-suspected patients visiting each selected health facility.

### 2.4. Data Collection Tools and Technique

Pretested structured interviewer-administered questionnaires were used to collect the data. Sputum samples were collected from patients who had productive cough of two weeks and above. During collection, two consecutive sputum samples at least 5-10 ml were collected in clean, sterile, leak-proof, screw-caped, wide-mouthed, disposable containers. Two consecutive sputum samples (spot-spot) were used to determine the sputum smear PTB positivity among TB-suspected patients. Simultaneously, records of TB suspects were reviewed from laboratory TB registration books to crosscheck sputum smear findings. One laboratory technologist supervisor and four laboratory technicians (data collectors) participated in data collection activities after taking a two-day training. Data collectors performed exit interviews on TB-suspected patients and reviewed TB laboratory registration logbooks for previous slide results. The two sputum samples per TB suspect were processed using the acid-fast bacilli staining technique and examined through an Olympus microscope. GeneXpert was used at the same time to compare the results of the two tests.

The sample was processed by Bsc laboratory technician. Sputum source was the site from which sputum was collected (mouth or lung), and it was evaluated through observation of patients while collecting sputum samples. Staining quality was defined as processing the smearing and staining steps as per the WHO staining standards. Sputum volume was the amount of sputum sample collected as per the WHO standard (minimum of 2-4 ml) required to process [12].

### 2.5. Data Quality Assurance

Data collectors and supervisors took a two-day training on study objectives, data collection procedures, and data confidentiality prior to actual data collection. Before conducting the actual study, the questionnaire was pilot-tested at nearby two health institutions for 10 patients with signs and symptoms of tuberculosis. Conducting supportive supervision of data collectors, processing, and reading of leftover sputum was as per WHO/national TB processing guidelines. Rereading of sputum smears by senior laboratory technicians who had more experience with TB slide reading and frequent manual and computer data editing were performed to assure data quality.

### 2.6. Data Management and Analysis

Data were entered, edited, and analyzed using the SPSS version 25. A descriptive analysis was performed on the demographic and clinical data to describe study objectives. Bivariate logistic regression was used to identify the association between smear positivity and the independent variable. The variables whose significance level was *p* < 0.2 were considered as candidates for the multivariable logistic regression analysis using enter method. Multicollinearity test was done among the independent variables using variance inflation factor (VIF), and no significant (VIF > 10) collinearity was detected. A model goodness-of-fit was checked by a nonstatistical result of the Hosmer-Lemeshow test (*p* = 0.64), which showed that the model was fitted to the data well. The association between sputum smear positivity and independent variables was declared as statistically significant for *p* values < 0.05.

## 3. Results

### 3.1. Sociodemographic Characteristics of Participants with the Prevalence of Smear PTB+

A total of 220 PTB-suspected patients participated in this study. The mean age of the study participants was 35.7 (SD = 15.7) years. The oldest study participant was 96 years. Among the study participants, the majority of them were in the age range of 18-35 years, and one hundred nineteen (54.1%) of them were males. Of the total study participants, one hundred sixty (72.7%) of them were married. One hundred thirty-four (60.9%) study participants were rural residents, and fifty-five (25.0%) were employed either at government or nongovernmental offices. By religion, 157 (71.4%) were protestant. Sixty-two (28.2%) respondents were illiterate.

In this study from the total of 220 sputum samples, 105 (52.5%) were positive for TB, of which 40 (18.1%) were AFB smear positive and 65 (61.9%) were GeneXpert MTB/RIF assay positive. The proportion of smear positivity was higher: 21 (18.8%) among females and 20 (21.7%) in the age group of 18-29 years (Table 1).

Based on bivariable logistic regression analysis, there was no sociodemographic factor significantly associated with bacteriologically confirmed PTB positivity (Table 1).

### 3.2. Behavioural- and Clinical-Related Characteristics of Suspected TB Patients with Smear-Positive PTB

The majority of patients came with a combination of symptoms and had chronic cough for more than two weeks. The most frequently reported symptoms were production of sputum 208 (94.5%) and chronic cough 196 (89.1%). Of the study participants, 43 (19.5%) had contact history with known positive PTB cases. Among the study participants, 25 (11.4%) had abnormal chest X-ray (Table 2).

From the bivariable logistic regression analysis, contact history with a suspected TB patient, smoking cigarette, and previously treated for TB were significantly associated with bacteriologically confirmed smear-positive TB (Table 2).

### 3.3. Factors Associated with Smear-Positive PTB

From the multivariable logistic regression analysis, contact history to a TB-suspected patient, smoking cigarette, and production of sputum/cough were statistically associated with smear-positive pulmonary tuberculosis. Respondents who had previous history of contact with a tuberculosis patient were about 2.48 times (*AOR* = 2.48, 95%*C*.*I* = 1.47, 5.23) more likely to develop smear-positive pulmonary tuberculosis than those who had no history of contact. Respondents who smoked cigarettes were 1.14 times more likely to be TB smear positive compared to respondents who did not smoke (Table 3).

## 4. Discussion

In this study, a health facility-based prevalence of PTB is reported using smear microscopy. The sputum smear positivity rate of PTB was found to be 18.2% (1,820 per 10,000). This clearly indicated that despite the measures that have been taken, tuberculosis remains a public health problem.

This finding was somewhat higher compared to study findings from Ghana (13%) [13], India (1.15%) [14], South Africa (3.9%) [15], and Botswana (4.2%) [16]. But they were lower than the study reports done in Pakistan (28.3%) [17], Peru (34%) [18], and Zambia (28%) [19]. These differences may include variations in population awareness, access to information, educational attainment, and strength of DOTs.

This finding was also higher compared to other study findings done in Ethiopia: Arsi Zone (7.3%) [20], Metehara Sugar Factory Hospital in Eastern Ethiopia (14.2%) [21], Dessie Referral Hospital (6.2%) [22], Northwest Ethiopia (4.9%) [11], and Western Amhara (12.8%) [23]. However, this finding was lower than in the previous study findings done in different parts of Ethiopia: Gamo Gofa (19.4%) [8], Eastern Ethiopia (21.0%) [24], and Bahir Dar health center (21.3%) [25]. This may reflect the differences in the study setups (private vs. only public), community participation, study area (rural vs. urban), and community awareness.

In this study, the high prevalence rate of smear-positive pulmonary TB was observed among males (54.1%) compared to females (45.9%). This result was found similar to the pattern shown in the study conducted in India and Uganda [26, 27]. The high prevalence rate observed among males might be due to the fact that men have greater social connections with other people and greater risk of exposure to persons with TB disease, thus having a higher chance of becoming infected with TB.

In this study, sociodemographic factors did not show a statically significant association with smear-positive pulmonary tuberculosis; this was somewhat similar with the study done in Addis Ababa and Nekemte Referral Hospital, Western Ethiopia [28, 29].

This study revealed that history of previous contact to PTB patients was one of the risk factors for PTB, which is consistent with the study conducted at Seka Health Center, Oromia Region, and in Nekemte Referral Hospital, Western Ethiopia [29, 30].

The role of smoking is well understood in the development of TB [31]. In this study, respondents who smoke cigarettes were significantly associated with smear positive compared to respondents who did not smoke. This result is consistent with other studies done in South East Ethiopia [32].

Thus, using MTB culture as the reference standard, this study compared the performance of the sputum GeneXpert MTB/RIF assay with traditional sputum AFB staining in patients with clinically suspected pulmonary TB. In this study based on sputum GeneXpert MTB/RIF assay, the prevalence of smear-positive TB was 65 (29.5%), which was higher than another study done in Nekemte Hospital [29] where the prevalence rate of smear-positive PTB was 15.88%.

## 5. Limitation of the Study

The study was conducted with small sample size, and some laboratory characteristics for diagnosis of smear-positive pulmonary TB were not included.

## 6. Conclusions

The prevalence of smear-positive pulmonary TB in Gedeo Zone health facilities was 18.2%, which is significantly high, and the MTB detection rate of GeneXpert was 29.5%. The risk factors significantly associated to this prevalence rate were contact with a TB patient, smoking cigarettes, and previously treated for TB. We recommend intervention on the identified associated risk factors and further studies to ascertain the risk factors and magnitude at the community level. Furthermore, a large-scale triangulated study is needed to identify sociodemographic factors and why PTB continues to be a major public health problem in Ethiopia in spite of the presence of TB program interventions.

## Figures and Tables

**Table 1:** Sociodemographic characteristics of TB-suspected patients with smear-positive PTB and prevalence.

Variable	Response	Total (*N*, %)	Smear-positive PTB		COR (95% CI)	*p* value
			-ve (*n*, %)	+ve (*n*, %)		
Sex	Male	119 (54.1)	98 (82.4)	21 (17.6)	1.08 (0.54, 2.15)	0.82
	Female	101 (45.9)	82 (81.2)	19 (18.8)	1	

Age	18-29	92 (42.0)	72 (78.3)	20 (21.7)	1	
	30-39	48 (29.1)	40 (83.3)	8 (16.7)	1.8 (0.54, 6.05)	0.34
	40-49	33 (15.1)	27 (81.8)	6 (18.2)	1.20 (0.38, 3.74)	0.75
	≥50	46 (21.0)	40 (87.0)	6 (13)	0.86 (0.21, 3.56)	0.84

Place of residence	Urban	86 (39.1)	72 (83.7)	14 (16.3)	0.81 (0.39, 1.65)	0.56
	Rural	134 (60.9)	108 (80.6)	26 (19.4)	1	

Marital status	Single	60 (27.3)	47 (78.3)	13 (21.7)	1	
	Married	160 (72.7)	133 (83.1)	27 (16.9)	1.36 (0.65, 2.86)	0.41

Educational status	No education	62 (28.2)	52 (83.9)	10 (16.1)	1	
	Primary	92 (41.8)	73 (79.3)	19 (20.7)	1.41 (0.35, 5.62)	0.63
	Secondary	41 (18.6)	33 (80.5)	8 (19.5)	1.92 (0.52, 7.06)	0.33
	College/university	25 (11.4)	22 (88.0)	3 (12.0)	1.78 (0.42, 7.45)	0.43

Religion	Orthodox	49 (22.3)	41 (83.7)	8 (16.3)	1	
	Protestant	157 (71.4)	127 (80.9)	30 (19.1)	1.17 (0.22, 6.27)	0.85
	Muslim	14 (87.7)	12 (85.7)	2 (14.3)	1.42 (0.30, 6.67)	0.66

Occupation	Government employee	33 (15.0)	29 (87.9)	4 (12.1)	1	
	Farmer	55 (25)	41 (74.5)	14 (25.5)	0.89 (0.23, 3.74)	0.87
	Merchant	17 (7.7)	15 (88.2)	2 (11.8)	2.22 (0.77, 6.35)	0.13
	Daily labor	26 (11.8)	21 (80.8)	5 (19.2)	0.86 (0.15, 4.78)	0.87
	Student	44 (20.0)	35 (79.5)	9 (20.5)	1.54 (0.42, 5.67)	0.51
	Housewife	45 (20.5)	39 (86.7)	6 (13.3)	1.67 (0.54, 5.17)	0.37

HH monthly income	<2000	79 (35.9)	60 (75.9)	19 (24.1)	1	
	2001-7000	131 (59.5)	110 (84.0)	21 (16.0)	5.59 (0.69, 14.6)	0.11
	≥7001	13 (4.5)	8 (85.2)	5 (14.8)	3.24 (0.41, 12.73)	0.24

**Table 2:** Behavioural- and clinical-related characteristics of PTB-suspected patients.

Variable	Response	Total (*N*, %)	Smear-positive PTB		COR (95% CI)	*p* value
			Yes (*n*, %)	No (*n*, %)		
Contact with suspected TB patients	Yes	43 (19.5)	17 (39.5)	26 (60.5)	3.23 (1.12, 4.48)	0.001
	No	177 (80.5)	23 (13.0)	154 (87.0)	1	
Vaccinate for TB	Yes	28 (12.7)	35 (18.2)	23 (82.1)	1.03 (0.36, 2.88)	0.96
	No	192 (87.3)	5 (17.9)	157 (81.8)	1	
Smoking cigarette	Yes	5 (2.3)	3 (60.0)	2 (40.0)	1.36 (1.02, 3.86)	0.03
	No	215 (97.7)	37 (17.2)	178 (82.1)	1	
Drinking alcohol	Yes	46 (20.9)	8 (17.4)	38 (82.6)	1.07 (0.45, 2.51)	0.87
	No	174 (78.1)	32 (18.4)	142 (81.6)	1	
Drink raw milk	Yes	149 (67.7)	26 (17.4)	123 (82.6)	1.16 (0.56, 2.39)	0.68
	No	71 (32.3)	14 (19.7)	57 (80.3)	1	
Chew chat	Yes	26 (11.8)	5 (19.2)	21 (80.8)	0.92 (0.32, 2.62)	0.88
	No	194 (88.2)	35 (18.0)	159 (82.0)	1	
HIV serostatus	Negative	203 (92.3)	37 (18.2)	166 (81.8)	0.96 (0.26, 3.52)	0.95
	Positive	17 (7.7)	3 (17.6)	14 (82.4)	1	
Previously treated for TB	Yes	47 (21.4)	12 (25.5)	35 (74.5)	0.56 (0.26, 1.22)	0.01
	No	173 (78.6)	28 (16.2)	145 (83.5)	1	
Chronic cough (>2 weeks)	Yes	196 (89.1)	161 (82.1)	35 (17.9)	0.83 (0.28, 2.36)	0.04
	No	24 (10.9)	19 (79.2)	5 (20.8)	1	
Production of sputum/cough	Yes	208 (94.5)	38 (18.3)	170 (81.7)	0.71 (1.06, 0.58)	0.04
	No	12 (5.5)	2 (16.7)	10 (83.3)	1	
CXR normal	Yes	195 (88.6)	38 (19.5)	157 (80.5)	2.78 (0.63, 12.3)	0.18
	No	25 (11.4)	2 (8.0)	23 (92.0)	1	

**Table 3:** Multivariable analysis of risk factors significantly associated with smear-positive PTB.

Variable	Response	Smear-positive PTB		AOR (95% CI)	*p* value
		Positive (%)	Negative (%)		
Contact to a TB patient	Yes	17 (39.5)	26 (60.5)	2.48 (1.47, 5.23)	0.001^∗∗^
	No	23 (13.0)	154 (87.0)	1	
Smoking cigarette	Yes	3 (60.0)	2 (40.0)	1.14 (0.2, 2.98)	0.003^∗∗^
	No	37 (17.2)	178 (82.1)	1	
Previously treated for TB	Yes	12 (25.5)	35 (74.5)	0.91 (0.35, 2.35)	0.09
	No	28 (16.2)	145 (83.5)	1	
Chronic cough (>two weeks)	Yes	161 (82.1)	35 (17.9)	0.73 (0.22, 0.94)	0.05^∗^
	No	19 (79.2)	5 (20.8)	1	
Production of sputum/cough	Yes	38 (18.3)	170 (81.7)	0.71 (1.06, 0.58)	0.04^∗^
	No	2 (16.7)	10 (83.3)	1	

Key: ^∗^ = significantly associated; ^∗∗^ = strongly associated; 1 = reference.

## Data Availability

Datasets used and/or analyzed during the current study are available from the corresponding author on reasonable request.

## Abbreviations

AFB: Acid-fast bacilli
AIDS: Acquired immunodeficiency syndrome
AOR: Adjusted odds ratio
BCTB: Bacteriologically confirmed tuberculosis
CI: Confidence interval
CKD: Chronic kidney disease
COR: Crude odds ratio
DM: Diabetes mellitus
DOTS: Directly observed treatment short-course chemotherapy
PTB: Pulmonary tuberculosis
SI: Severe immunosuppression
TB: Tuberculosis
TMR: Tuberculosis mortality rate
WHO: World Health Organization.
